# Hummingbird community structure and nectar resources modulate the response of interspecific competition to forest conversion

**DOI:** 10.1007/s00442-023-05330-z

**Published:** 2023-02-09

**Authors:** Esteban A. Guevara, Carolina Bello, Cristian Poveda, Ian R. McFadden, Matthias Schleuning, Loïc Pellissier, Catherine H. Graham

**Affiliations:** 1 Biodiversity and Conservation Biology, Swiss Federal Research Institute WSL, Zürcherstrasse 111, 8903 Birmensdorf, Switzerland; 2 Área de Investigación y Monitoreo de Avifauna, Aves y Conservación, BirdLife in Ecuador, Nuño de Valderrama OE7 y Av, Mariana de Jesús, Quito, Ecuador; 3 Landscape Ecology Group, Department of Environmental Systems Science, Institute of Terrestrial Ecosystems, ETH Zürich, 8092 Zurich, Switzerland; 4 Senckenberg Biodiversity and Climate Research Centre (SBiK-F), Senckenberganlage 25, Main, 60325 Frankfurt am Main, Germany

**Keywords:** Andes, Context dependency, Ecuador, Land-use change, Morphological dissimilarity, Selectivity

## Abstract

On-going land-use change has profound impacts on biodiversity by filtering species that cannot survive in disturbed landscapes and potentially altering biotic interactions. In particular, how land-use change reshapes biotic interactions remains an open question. Here, we used selectivity experiments with nectar feeders in natural and converted forests to test the direct and indirect effects of land-use change on resource competition in Andean hummingbirds along an elevational gradient. Selectivity was defined as the time hummingbirds spent at high resource feeders when feeders with both low and high resource values were offered in the presence of other hummingbird species. Selectivity approximates the outcome of interspecific competition (i.e., the resource intake across competing species); in the absence of competition, birds should exhibit higher selectivity. We evaluated the indirect effect of forest conversion on selectivity, as mediated by morphological dissimilarity and flower resource abundance, using structural equation models. We found that forest conversion influenced selectivity at low and mid-elevations, but the influence of morphological dissimilarity and resource availability on selectivity varied between these elevations. At mid-elevation, selectivity was more influenced by the presence of morphologically similar competitors than by resource abundance while at low-elevation resource abundance was a more important predictor of selectivity. Our results suggest that selectivity is influenced by forest conversion, but that the drivers of these changes vary across elevation, highlighting the importance of considering context-dependent variation in the composition of resources and competitors when studying competition.

**Supplementary Information:**

The online version contains supplementary material available at 10.1007/s00442-023-05330-z.

## Introduction

Land-use change influences not only species diversity, but also the ecological mechanisms that promote species co-existence (Tylianakis et al. 2008; Schmeller et al. 2018). Specifically, land-use change imposes constraints on species assemblages directly, via environmental filtering by modifying natural abiotic conditions of ecosystems such as temperature or light (Jakovac et al. 2016; Schmeller et al. 2018), and indirectly, by altering biotic interactions (Valladares et al. 2015). Interspecific competition, in particular, is recognized as an important mechanism influencing the maintenance of biodiversity (MacArthur and Levins 1967; Colwell 1973; Jankowski et al. 2010; Pigot and Tobias 2013; Grether et al. 2017); therefore, it is critical to understand how it is influenced by land-use change. Here, we conducted an experimental study along an elevation gradient in the Andes of Ecuador to evaluate the direct and indirect effects of land-use change (i.e., forest conversion) on interspecific competition in tropical hummingbirds.

Species with similar morphological traits are more likely to compete for the same resources and are less likely to co-exist (Tilman 1982; Chesson 2000; Calizza et al. 2017); therefore, niche differentiation often manifests as morphological dissimilarity (Pyke 1982; Violle et al. 2011; Dehling et al. 2016). If species exploit different resources, and their preferred resources are readily available in the landscape, then competition may be reduced (Roeleke et al. 2018). However, if resources are limited, as it is sometimes the case in degraded habitats, competition may increase especially among morphologically similar species.

Investigating the relationship between forest conversion and biotic interactions is complex, since it may depend on the community context, i.e., the identity and abundance of the competing species and their resources (Quitián et al. 2018). For instance, organisms can switch from negative to positive interactions in different contexts of resource abundance and community composition (Kawai and Tokeshi 2007; Fugère et al. 2012). To address this complexity, selectivity experiments provide an opportunity to evaluate the outcome of interspecific competition when community composition varies (Pimm et al. 1985; Weinstein and Graham 2016). Selectivity is the time a species feeds at a high-value resource when presented both high- and low-value resources in the presence of competing individuals from different species (Pimm et al. 1985). Selectivity should be high when the energetic gain of feeding at the high-value resource is higher than the cost of defending this resource (Carpenter 1987; Temeles et al. 2002; Salvidio et al. 2012; Weinstein and Graham 2016). The cost of defending a resource against species with similar morphologies may be particularly high, resulting in low selectivity. Alternatively, selectivity could be high if alternative resource levels are high, and these resources can be exploited at a low cost. Selectivity approximates the outcome of interspecific competition, because it measures the resource intake across competing species (Sandlin 2000).

Hummingbirds are an ideal system to study the relationship between forest conversion and selectivity, because the composition of hummingbird assemblages changes with forest disturbance (Tinoco et al. 2018) and hummingbirds strongly compete for nectar resources (Stiles 1975; Feinsinger and Colwell 1978). Furthermore, the assemblage structure of hummingbirds varies across elevation gradients in the Andes (Graham et al. 2012; Lessard et al. 2015), allowing the evaluation of forest conversion effects on competition within assemblages with different composition. At high elevations, co-occurring species exhibit more limited trait variation than at lower elevations due to environmental filtering (Graham et al. 2009) and higher evenness in morphological space (Graham et al. 2012; Lessard et al. 2015). In contrast, at mid-to-low elevations, morphological traits exhibit a greater range of variation (Graham et al. 2012; Lessard et al. 2015), and morphological spacing is random (compared to the regional species pool). In addition, hummingbird species with large body sizes, long bills, and specialized diets are generally more sensitive to forest conversion, leading to homogenized and generalist communities in disturbed environments (Tinoco et al. 2017; 2018).

**Fig. 1:**
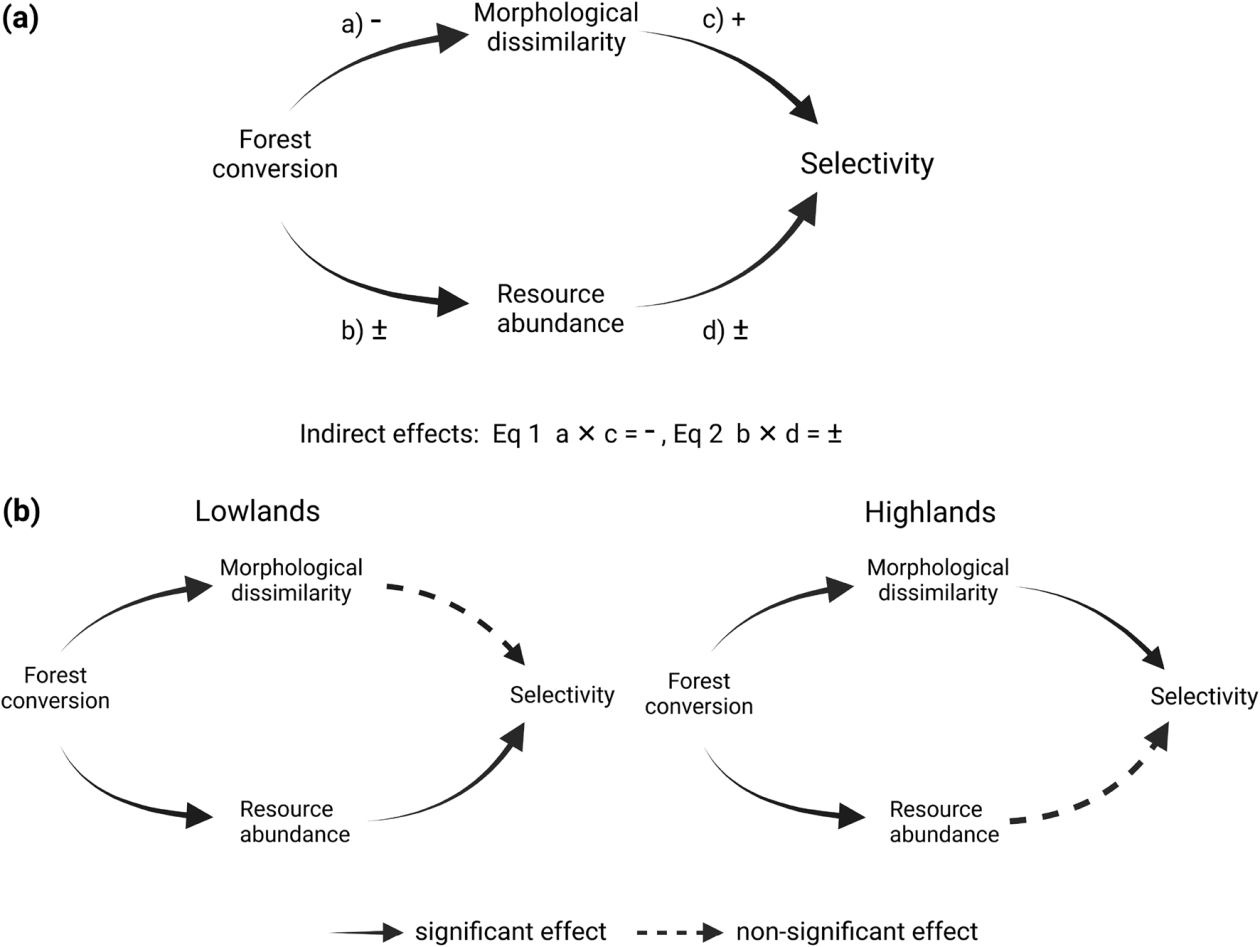
**a** General model of the indirect effect of forest conversion on selectivity mediated by morphological dissimilarity and resources. The plus and minus signs indicate the direction of the relationships. Indirect relationships between forest conversion and selectivity are hypothesized as the product of regression slopes of direct relationships (bottom left equations). b Expected effect of elevation on the contribution of morphological dissimilarity and resources in explaining selectivity

Here, we experimentally tested the indirect effects of forest conversion on hummingbird selectivity along an elevational gradient in the Andes of Ecuador. We assessed the influence of forest conversion on hummingbird selectivity, mediated by morphological dissimilarity and resource abundance (Fig. 1), at three different elevations and asked whether: (1) forest conversion influences hummingbirds’ morphological dissimilarity and flower abundance; (2) morphological dissimilarity and flower abundance correlate with selectivity; and (3) differences in hummingbird assemblage composition influence the indirect effect of forest conversion on selectivity, mediated by morphological dissimilarity and resources. We expected that: (1) forest conversion will decrease hummingbirds’ morphological dissimilarity (Fig. 1a, path *a*) and alter resource abundance (Fig. 1a, path *b*). (2) When morphological dissimilarity with co-occurring species is high, we hypothesize that selectivity will increase (Fig. 1a, path *c*). Therefore, with forest conversion species become more similar, which might result in greater competition and lower selectivity (Fig. 1a, Eq. 1). The relationship between selectivity and resources could be positive or negative. For instance, as resources increase, hummingbirds may become more selective, because competition will be lower, given that they have alternative resources in the landscape (Weinstein and Graham 2016). Alternatively, when resources are low, species may more aggressively defend the high-quality feeder yielding a negative relationship between selectivity and resource abundance (Fig. 1a path *d*). With forest conversion, resources could either increase or decrease resulting in either higher or lower selectivity (Fig. 1a, Eqs. 2, 3). Finally, at high elevation, morphological dissimilarity may have a stronger positive influence on selectivity (Fig. 1b, ‘Highlands’), as morphological variation is limited (Graham et al. 2012), while at low elevation, where species have more variable morphology, resources may have a greater influence on selectivity (Fig. 1b, ‘Lowlands’).

## Materials and methods

### Study area and design

Our study area is located in the northwestern Andes of Ecuador, within Pichincha province, along an elevation gradient ranging from 800 to 3500 m a.s.l. This area, which is part of the Tropical Andes biodiversity hotspot, has one of the highest levels of diversity and endemism in the world, as well as high rates of deforestation (Myers et al. 2000). The average annual precipitation is ~ 2000 mm, with a dry season between July to September when precipitation drops to approximately 100 mm (Mordecai et al. 2009). We consider two types of habitats, forest and converted areas. Forest refers to large tracks of mature or old second-growth forest, whereas converted areas are cattle farms characterized by remnants of forest embedded within a pasture matrix (i.e., cattle farms dominated by pastures with hedgerows, live fences, and small forest patches embedded). We used a block design (Feinsinger 2003) to establish 18 experimental trials spread across three blocks at low (800 m a.s.l), mid (2200 m a.s.l), and high (3500 m a.s.l) elevations, respectively. In each block, six trials were conducted, three in forest and three in converted areas. We placed experimental trials in areas surrounded by vegetation to reduce predation risk of foraging hummingbirds (Carthey and Banks 2015). In converted areas, we therefore avoided completely cleared areas and placed experimental trials within remnants of forest vegetation (e.g., small forest patches or hedgerows). This placement of feeders, within remaining forest vegetation in deforested areas, allowed us to quantify flower abundance nearby experimental trials. The minimum distance between trials was 300 m, and coordinates and elevation of each trial are provided in Table S1. During each trial, we gathered data on plant and hummingbird composition, flower abundance, and conducted selectivity experiments. An overview of the study design and the spatial placement of experimental trials is provided in Figs. S1 and S2, respectively.

### Quantification of flower resources and hummingbird morphological dissimilarity

To relate resources and morphological dissimilarity with selectivity, our proxy for interspecific competition (described below), we quantified flower abundance and hummingbird composition in the 18 trials. We counted all open flowers in the vicinity (100 m) of each trial and within 5 m on each side of the trail. This quantification was done simultaneously to selectivity experiments. This effort included plants identified as being used by hummingbirds based on Weinstein and Graham (2016), and in video sampling of flowers conducted over 2 years (Guevara et al. in review). We included both flowers that exhibit a bird-adapted syndrome, i.e., elongated with bright-colored corollas (Fægri and van der Pijl 1979; Hingston and McQuillan 2000) and those less likely used by hummingbirds (e.g., white and short open corollas). For flowers that were too numerous to count directly, we estimated the total number of flowers by randomly choosing five flower units (e.g., inflorescences, stalks, or flower aggregations) and multiplying the average number of flowers in these five units by the total number of units in the plant. To explore possible temporal variation in resource abundance across sites, we included information of a different study where repeated flower counts were done on a bimonthly basis (i.e., every 2 months) from December 2017 to January 2020 (Guevara et al. in review), and results from this census are presented in Fig. S3.

To quantify morphological dissimilarity, we compiled a hummingbird species list obtained from video sampling at feeders (see below) and at flowers adjacent to experimental trials (Guevara et al. in review). We did this, since not all hummingbirds might be attracted to feeders, although present in the area (Ramírez-Burbano et al. 2022). Morphological dissimilarity of hummingbirds was based on body mass, bill length, and tarsus length (Graham et al. 2012). Body mass influences thermoregulation and species’ ability to defend high rewarding resources (Altshuler et al. 2004; González-Gómez et al. 2011); thus, larger hummingbirds are better suited to defend resources (Altshuler 2006). Bill length is associated with resource use and foraging efficiency (Feinsinger and Colwell 1978; Temeles et al. 2002; Maglianesi et al. 2015); hummingbirds with very long bills could experience increased handling costs at short-corolla flowers or feeders (Maglianesi et al. 2015) which could influence selectivity. Tarsus length is related to perching ability and territoriality on feeding patches (Stiles 2004). To quantify morphological dissimilarity, we first performed a principal component analysis (PCA) for each habitat/elevation assemblage using the values of the morphological traits described above. We quantified the assemblage centroids by averaging the scores of each species along the first two principal components that summarized in average 97% of morphological variation. We then calculated the Euclidean distance of species scores to the assemblage centroids as our measure of morphological dissimilarity (i.e., how different is a species compared to the assemblage). To account for a possible influence of activity levels at feeders on morphological dissimilarity (i.e., common species at feeders would exhibit low morphological dissimilarity among con-specific individuals), we calculated activity levels for each species, in each site and habitat. Activity levels was calculated as the number of visits of a given species divided by the number of visits of all species. Then, we weighted the morphological centroids by activity levels, so the centroids shifted toward more active species at feeders. Morphological dissimilarity was then recalculated relative to the activity-weighted centroid. This additional measure of morphological dissimilarity was used in a sensitivity test (see data analyses).

### Selectivity experiments

Before starting the experiments, we left feeders filled with sucrose solution in the field for 3 days, so birds could detect and acclimate to using them (Weinstein and Graham 2016). The feeders were cleaned, and the nectar replenished on alternative days. After the acclimation period, we conducted experiments in three replicated trials, totaling 18 experimental trials (i.e., 3 trials per habitat × 2 habitats per block × 3 blocks, Fig. S1). Each trial consisted of a pair of feeders, placed at c. 2 m height and 8–10 m apart from each other, a distance close enough to allow a hummingbird to detect both feeders, but far enough to discourage a single bird from defending both simultaneously (also see Weinstein and Graham 2016). Each trial had a feeder with a high (30% sucrose) and low (10% sucrose) sucrose concentration. This range of concentrations corresponds to values reported for flowers found in the region (Weinstein and Graham 2016) and hummingbird-visited plants in general (Altshuler 2006; Baker 1975). The location of the high- and low-concentration feeders was switched after the third day of observations, and then another 3 days of observations were made. Experimental trials were performed simultaneously at forest and converted transects. The dates at which each experimental trial was performed are detailed in Table S1. For simultaneous observations at each feeder, we used time-lapse cameras placed in front of feeders, which recorded hummingbird visits between 06h00 and 18h30. We analyzed videos with Deep Meerkat v 0.0.9 (Weinstein 2018). Deep Meerkat is an image classification software based on a convolutional neural network algorithm that discriminates video frames with potential animal activity from those with no animal activity (Weinstein 2018). Once the program selected the frames with hummingbird activity, we manually reviewed them to identify species (Fig. S4). We quantified selectivity for every species and for every hour in a filming day by dividing the time spent feeding on the high-value resource by the total feeding time at both feeders (Pimm et al. 1985; Sandlin 2000; Weinstein and Graham 2016). Interspecific competition in animals is often quantified as the outcome of interspecific aggression or interference competition (displacement or individual chases; Temeles et al. 2002; Mac Nally and Timewell 2005; Gibb and Johansson 2011). Instead, we chose selectivity as measure of exploitative competition, since it is often unclear what constitutes a successful chase, and whether “successful” hummingbirds ultimately gain energetic benefits from these aggressive interactions, i.e., feeding at the high-value feeder.

### Data analysis

Given the possible variation in resource abundance and hummingbird assemblage composition across elevations (Graham et al. 2012; Lessard et al. 2015), we performed independent analyses for each elevation by subsetting our dataset in three elevational bins (i.e., low, mid, and high). We summarized our data by extracting the mean values of selectivity for each species, and this step was done for each trial, habitat, and day of sampling combination (*N* = 104 selectivity measures for high elevation, and *N* = 103 for each mid and low elevations). To account for possible temporal autocorrelation due to sampling selectivity over consecutive days, in the subsequent tests, we applied linear mixed models (lmm) with day of sampling as a random effect. To obtain standardized parameter estimates and ensure comparability among models, we scaled all variables to mean = 0 and standard deviation = 0.5 (Fan et al. 2016).

We defined relationships among selectivity and explanatory variables according to a general piecewise structural equation model (SEM, Fig. 1a). In this model, forest conversion was defined as a dummy variable (0 = forest, 1 = converted), and we tested its influence on resources and morphological dissimilarity (question 1, Fig. 1). In the same model, we tested the relationship between morphological dissimilarity and resource abundance to selectivity (question 2). To estimate the indirect effect of forest conversion on selectivity, we multiplied the regression coefficients of the equations defined in SEM (Fig. 1a, Fan et al. 2016), as follows: (1) paths *a* × *c* (indirect effect mediated by morphological similarity), and (2) paths *b* × *d* (indirect effect mediated by resource abundance). To assess whether differences in assemblage composition along the elevation gradient influence the response of selectivity to forest conversion (question 3), we visually compared the direction and magnitude of the parameters estimated in our SEM models for the three elevations. To account for a possible influence of differences on activity levels across species on selectivity (i.e., more active species at feeders would compete more with con-specifics and exhibit lower selectivity), we repeated the analysis using our activity-weighted measure of morphological dissimilarity. These results did not differ qualitatively from our first analysis and are presented in supplementary material Table S6.

Fisher’s *C* statistic was used to assess the global goodness of fit of our SEMs, values of *p* > 0.05, which indicated that the hypothesized structure of the model is well supported by the data (Lefcheck 2016). For associations tested in our models, we calculated marginal and conditional R-squares values that describe the proportion of variance explained by both fixed and random effects, respectively (Nakagawa and Schielzeth 2013). Linear mixed and structural equation models were performed using packages lme4 (Bates et al. 2015) and piecewiseSEM (version 0.5-18) in R version 4.1.1 (R Core Team 2021).

## Results

We recorded 67 species of flowering plants (mean 13.67 ± 2.20 STD, range 11–18) in the trial counts. Mid-elevation trials had the highest richness of plants and hummingbirds, especially in the forest where both taxonomic groups exhibited the highest richness (Tables S2, S3). We detected 16 species of hummingbirds in cameras set at feeders during 108 days of filming (c. 1296 h). The influence of forest conversion on resource availability and morphological dissimilarity varied along the elevation gradient. Forest conversion positively influenced resource abundance at mid (*β* = 0.30, *P* < 0.01, *df* = 94) and high elevation (*β* = 0.5, *P* < 0.001, *df* = 15), and negatively at low elevation (*β* = − 0.77, *P* < 0.001, *df* = 75). Hummingbird morphological dissimilarity was not influenced by forest conversion at low (*β* = − 0.17, *P* = 0.10, *df* = 76) and high (*β* = − 0.05, *P* = 0.62, df = 15) elevations, but was negatively influenced at mid-elevation (*β* = − 0.40, *P* < 0.01, *df* = 94).

**Fig. 2:**
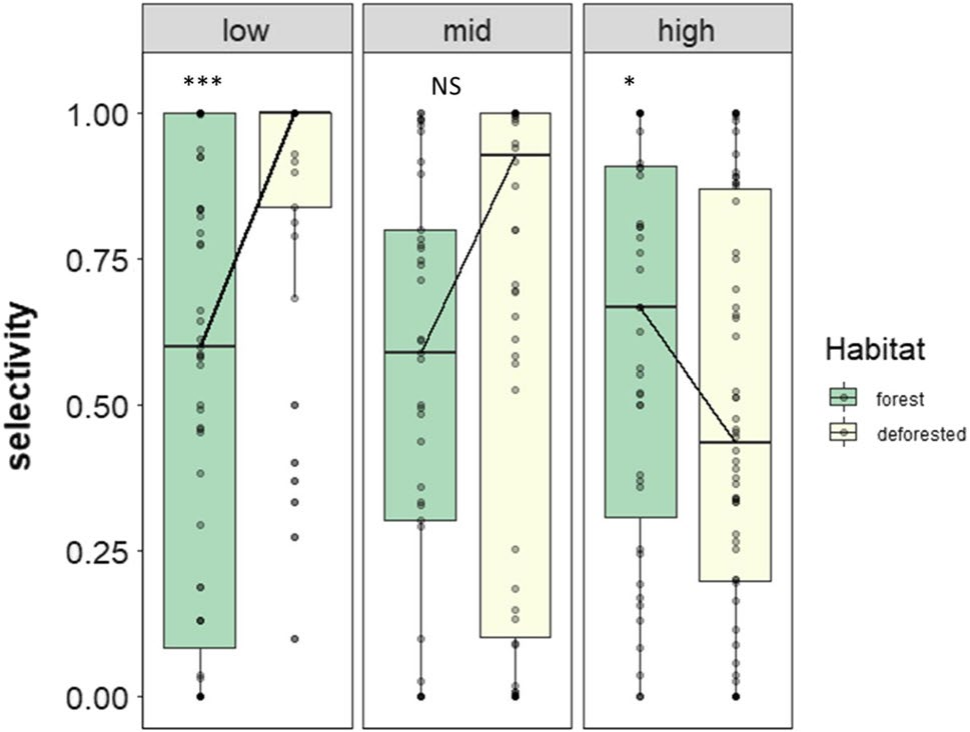
Differences in selectivity values between habitat types (forest and converted) at three different elevations along an elevation gradient of 800–3500 m. ***P < 0.001, *P < 0.05, *NS* non-significant differences

**Fig. 3:**
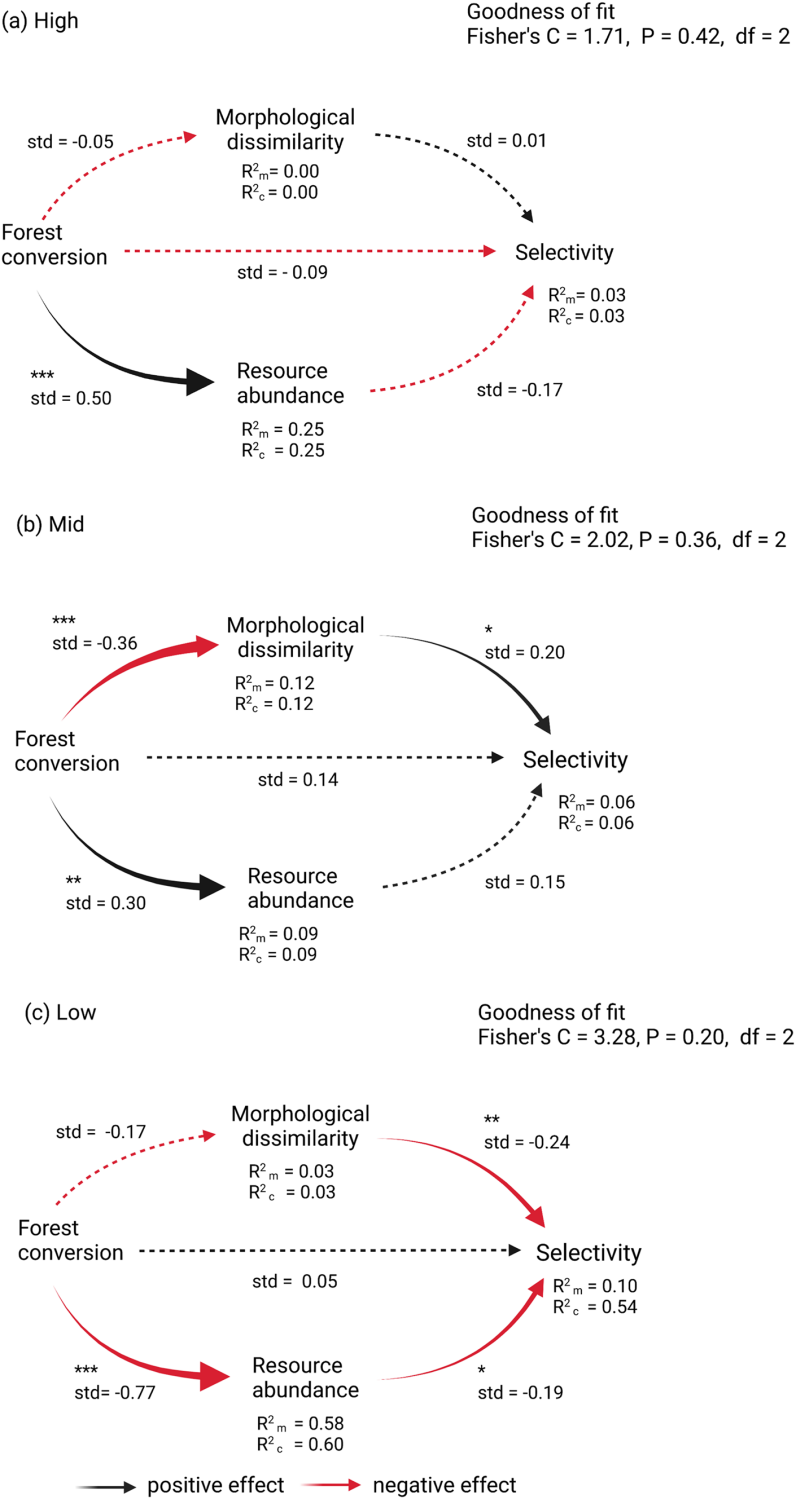
Relationships among forest conversion into pasture (0 = forest, 1 = converted), morphological dissimilarity, resource abundance, and selectivity for **a** high-elevation, **b** mid-elevation, and **c** low-elevation hummingbird communities. Standardized path coefficients (std) are shown; solid lines and asterisks indicate statistical significance (***P < 0.001, **P < 0.01, *P < 0.05). Provided are Fisher’s C goodness of fit as well as marginal (*R*_m_) and conditional (*R*_c_) R-squares. Marginal R-squares takes into account the variance for fixed effects only, whereas conditional R-squares takes into account variance of both fixed and random effects

Selectivity was slightly greater in the forest than in the converted transect at high elevation, but greater in the converted transect at low elevation (Fig. 2). As hypothesized, the importance of floral resources and morphological dissimilarity for explaining selectivity varied at different points of the elevation gradient (Fig. 3a–c). The indirect effect of forest conversion on selectivity was positive at low elevation and was mediated by a decrease in resource abundance (Fig. 3c, coefficient product = 0.15). In contrast, at mid-elevation, the indirect effect of forest conversion on selectivity was negative and was mediated by a decrease in morphological dissimilarity (Fig. 3b, coefficient product = 0.07). Neither morphology, nor resources explained variation in selectivity at high elevation. The direct effect of forest conversion on selectivity was not significant at the three elevations (Fig. 3a–c). The SEMs exhibited fair goodness of fit at low (Fisher’s *C* = 3.28, *P* = 0.2, *df* = 2), mid (Fisher’s *C* = 2.02, *P* = 0.36, *df* = 2), and high (Fisher’s *C* = 1.71, *P* = 0.42, *df* = 2) elevations. Models output is shown in Table S3.

## Discussion

Land-use change affects species composition, and hence, interactions among those species that occur in converted habitats (Tinoco et al. 2017; MacDougall et al. 2018; Quitián et al. 2018). Here, we found that forest conversion influences selectivity, our measure of interspecific competition, in Andean hummingbirds across an elevation gradient of 800–3500 m. Yet, the intensity and direction of this relationship depended on resource abundance and morphological dissimilarity among co-occurring species. At mid-elevation, selectivity was influenced by morphological dissimilarity among species in the clades of Coquettes and Brilliants (e.g., *Adelomyia*, *Coeligena, *and* Heliodoxa*)*,* while at low elevation, selectivity among species in the clades of Hermits and Emeralds (e.g., *Phaethornis*, *Amazilia*) was better explained by resource abundance. Our results demonstrate the importance of considering the context-dependent effect of land-use change on different types of biotic interactions, such as mutualisms (Tinoco et al. 2017; Quitián et al. 2018) and predator–prey interactions (MacDougall et al. 2018). Although context dependency can make it difficult to make generalizations about the effect of land use on biotic interactions, our results suggest that by considering community structure, we can develop and test expectations about how biotic interactions will respond to land-use change.

Our findings partially agree with previous research done in the study region (Weinstein and Graham 2016). For instance, we showed that the degree to which morphological dissimilarity and resource abundance influence selectivity varies with elevation, likely because of the different processes structuring community assembly along the elevation gradient. On the one hand, at higher elevations, hummingbird morphology tends to have a limited range and be evenly spaced in trait space, a pattern consistent with competition (Stiles 2004; Graham et al. 2012; Lessard et al. 2015). Our results support this possibility as we found that morphological differentiation predicts the degree of selectivity in mid-elevation hummingbirds, a result not found previously in a similar study (Weinstein and Graham 2016). On the other hand, previous studies report little evidence of trait evenness in the lowlands, suggesting that competition may have a more limited role in structuring these communities (Graham et al. 2012; Lessard et al. 2015). We show that selectivity in the lowlands is mostly driven by resource abundance rather than morphological dissimilarity. Thus, our experimental results are consistent with predictions from limiting similarity theory (MacArthur and Levins 1967).

Forest conversion resulted in a decreased hummingbird morphological dissimilarity at all elevations, although a significant effect was only found at mid-elevation, and increased resource abundance at high- and mid-elevations but decreased resource abundance at low elevation. Morphological dissimilarity in hummingbirds was lower in converted transects, a result consistent with previous studies where forest conversion filtered out species with high body mass and long bills (Hadley et al. 2018; Tinoco et al. 2018). Heavier hummingbirds might have smaller population sizes than lighter hummingbirds (Calder and Calder 1995), and thus may be more sensitive to habitat loss. In addition, hummingbirds with long bills tend to have a specialized diet (Wolf et al. 1972; Stiles 1975; Feinsinger and Colwell 1978; Maglianesi et al. 2015; Weinstein and Graham 2017). As a result, they may be less able to cope with changes in resource abundance resulting from forest conversion (Maglianesi et al. 2015; Tinoco et al. 2017). The effects of forest conversion on resources were mixed. On the one hand, increased availability of flowers at converted transects might be due to the release of critical environmental resources for successional plants (e.g., sunlight, soil nutrients; Blake and Hoppes 1986; Morellato et al. 2016; Pessoa et al. 2017). On the other hand, plant propagules are not able to colonize and survive in converted transects, and then, this will help to explain observed resource decrease at converted transects. Species turnover in plant communities along the elevation gradient may also contribute to the patterns observed in this study.

Whether it is because of resource scarcity or increased similarity, the influence of land-use change on the outcome of interspecific competition conforms with two of our expectations. First, we expected that forest conversion would result in a decrease of morphological dissimilarity and that competition among morphological similar species would yield higher selectivity if alternative resources available in the landscape could be exploited. This scenario was observed for mid-elevation hummingbirds. However, we cannot rule out the role of resources at this elevation, since we conducted experiments at different peaks of resource abundance between habitats (Fig. S3). Second, we predicted that forest conversion would reduce resources for hummingbirds forcing them to intensively compete for available resources, ultimately resulting in an increase in selectivity (Figs. 2, 3). Intensified competition could reduce diversity through competitive exclusion (Muthukrishnan et al. 2018) which could affect biodiversity at larger spatial scales.

### Implications for future research

In this study, we addressed external factors affecting species selectivity, but whether selectivity could also be affected by species abundance, behavior, or life history is yet to be determined. For instance, if a species is extremely common, then it would mostly compete with con-specifics, rendering interspecific competition less important. This may be the case at our high-elevation sites in forest habitat where a single species, *Coeligena lutetiae*, accounted for 79% of visits to experimental feeders (Table S5), suggesting that individuals of this species were mainly competing with con-specifics. Additionally, hummingbirds have different foraging behaviors in communities, including: territorial, defending fixed flower resources; generalists, feeding a broad array of resources opportunistically; and trap-lining, traveling to preferred resources sequentially throughout the day (Feinsinger and Colwell 1978). Here, we chose not to evaluate this behavioral trait, because, within species, this behavior can vary as a function of resources and competitors (Stiles and Wolf 1970; Stiles 1981) which in turn change across elevation. Since we compared assemblages at different elevations, our data are not suitable for relating foraging behavior with selectivity; however, this remains an open question to be best addressed with multiple replicates at a given elevation. In addition, life stages (breeding vs non-breeding, adult vs juvenile, and altitudinal migration) could affect the competitive ability of hummingbirds to attain optimal resources. Hummingbirds are known to balance their nitrogen needs by adding arthropods to their nectar-based diet, specially during the breeding season (Stiles 1995). A diminished dependence on nectar resources and shift to arthropods could affect dynamics of species interactions differently across life stages. Finally, foraging choices may also be influenced by predation risk (Breviglieri et al. 2013). In this sense, the giving-up density framework (Brown 1988), in which animals stop foraging at artificial resource patches when costs outweigh benefits, can be used to quantify predation risk perception (i.e., Breviglieri et al. 2013) as a factor influencing selectivity (Carthey and Banks 2015).

Our experimental approach offers a way to connect broad-scale processes, like environmental filtering, with local mechanisms such as competition and niche partitioning (Weber and Agrawal 2012; Weinstein and Graham 2016). Nonetheless, these experiments can be improved by collecting complementary data. First, independent observations on hummingbird abundance around experimental feeders (including birds not visiting them) could be used to help determine the influence of inter- and intra-specific differences on resource competition. Second, simultaneous observations of hummingbird visits to flowers and experimental feeders will help to get a clearer signal of the influence of resource abundance on species-level selectivity. Finally, advances in the deployment of tracking devices for light weighted birds (Williamson and Witt 2021) should allow us to evaluate selectivity at the individual level and link selectivity to the movements and breeding stage of individuals.

## Supplementary Information

Below is the link to the electronic supplementary material.

Supplementary file1 (DOCX 1470 KB)

## Data Availability

The dataset analyzed during the current study is available at: 10.6084/m9.figshare.21804336.
