# Supplementary material for: Hummingbird community structure and nectar resources modulate the response of interspecific competition to forest conversion
Source: Oecologia. 2023 Feb 9;201(3):761–70. doi: 10.1007/s00442-023-05330-z (PMC10038955; doi:10.1007/s00442-023-05330-z)
Supplement: Supplementary file 1 — Supplementary file1 (DOCX 1470 KB) [file 442_2023_5330_MOESM1_ESM.docx]

**SUPPORTING INFORMATION**

**Hummingbird community structure and nectar resources modulate the response of interspecific competition to forest conversion**

Esteban A. Guevara, Carolina Bello, Cristian Poveda, Ian R. McFadden, Matthias Schleuning, Loïc Pellissier and Catherine H. Graham

Table S1.- Coordinates and elevation of each study transect where flower counts and selectivity experiments were performed, dates when data were collected is also provided.

| Transect | Latitude | Longitude | Elevation range | Dates of experiments |
| --- | --- | --- | --- | --- |
| High forest | -0.12108 | -78.59030 | 3400-3500 | Dec 2018 |
| High converted | -0.10278 | -78.58757 | 3200-3400 | Oct 2018 |
| Medium forest | -0.00713 | -78.73633 | 1800-2000 | Jan 2019 |
| Medium converted | 0.01657 | -78.70180 | 1800-2000 | Nov 2018 |
| Low forest | 0.05217 | -78.84148 | 800-1200 | Sep 2018 |
| Low converted | 0.05599 | -78.83981 | 1100-1200 | Aug 2018 |

Table S2

Hummingbird food plant species recorded in transect counts (Guevara et al., 2023)

|  | Low | | | Mid | | | High | | |  |
| --- | --- | --- | --- | --- | --- | --- | --- | --- | --- | --- |
|  | | Forest | Deforested | | Forest | Deforested | | Forest | Deforested | |
| *Axinaea lehmannii* | | 0 | 0 | | 1 | 0 | | 0 | 0 | |
| *Barnadesia spinosa* | | 0 | 0 | | 0 | 0 | | 0 | 1 | |
| *Berberis paniculata* | | 0 | 0 | | 0 | 0 | | 0 | 1 | |
| *Besleria solanoides* | | 1 | 0 | | 0 | 0 | | 0 | 0 | |
| *Bomarea multiflora* | | 0 | 0 | | 0 | 0 | | 0 | 1 | |
| *Bomarea pardina* | | 0 | 0 | | 1 | 0 | | 0 | 0 | |
| *Brachyotum gleasonii* | | 0 | 0 | | 0 | 0 | | 1 | 1 | |
| *Brachyotum ledifolium* | | 0 | 0 | | 0 | 0 | | 0 | 1 | |
| *Brugmansia sanguinea* | | 0 | 0 | | 0 | 0 | | 0 | 1 | |
| *Burmeistera crispiloba* | | 0 | 0 | | 1 | 0 | | 0 | 0 | |
| *Calathea ischnosiphonoides* | | 0 | 0 | | 1 | 0 | | 0 | 0 | |
| *Calathea marantifolia* | | 1 | 1 | | 0 | 0 | | 0 | 0 | |
| *Centropogon nigricans* | | 0 | 0 | | 1 | 0 | | 0 | 0 | |
| *Centropogon pichinchensis* | | 0 | 0 | | 0 | 0 | | 1 | 0 | |
| *Centropogon solanifolius* | | 0 | 0 | | 0 | 1 | | 0 | 0 | |
| *Columnea angustata* | | 0 | 1 | | 0 | 0 | | 0 | 0 | |
| *Columnea ciliata* | | 0 | 0 | | 1 | 0 | | 0 | 0 | |
| *Columnea dielsii* | | 0 | 0 | | 0 | 0 | | 1 | 0 | |
| *Columnea eburnea* | | 1 | 1 | | 0 | 0 | | 0 | 0 | |
| *Columnea kucyniakii* | | 0 | 0 | | 1 | 0 | | 0 | 0 | |
| *Columnea medicinalis* | | 1 | 0 | | 1 | 1 | | 0 | 0 | |
| *Columnea spathulata* | | 1 | 1 | | 0 | 0 | | 0 | 0 | |
| *Costus pulverulentus* | | 1 | 0 | | 0 | 0 | | 0 | 0 | |
| *Drymonia turrialvae* | | 1 | 0 | | 0 | 0 | | 0 | 0 | |
| *Erythrina smithiana* | | 0 | 1 | | 0 | 0 | | 0 | 0 | |
| *Fuchsia ayavacensis* | | 0 | 0 | | 0 | 0 | | 1 | 1 | |
| *Gasteranthus glaber* | | 0 | 0 | | 1 | 0 | | 0 | 0 | |
| *Gasteranthus pansamalanus* | | 1 | 0 | | 0 | 0 | | 0 | 0 | |
| *Gasteranthus quitensis* | | 1 | 0 | | 0 | 1 | | 0 | 0 | |
| *Gaultheria insipida* | | 0 | 0 | | 0 | 0 | | 1 | 0 | |
| *Glossoloma herthae* | | 0 | 0 | | 1 | 0 | | 0 | 0 | |
| *Glossoloma sprucei* | | 1 | 0 | | 0 | 0 | | 0 | 0 | |
| *Glossoloma subglabrum* | | 0 | 0 | | 1 | 0 | | 0 | 0 | |
| *Guzmania angustifolia* | | 0 | 1 | | 0 | 0 | | 0 | 0 | |
| *Guzmania jaramilloi* | | 0 | 0 | | 1 | 0 | | 0 | 0 | |
| *Guzmania teuscheri* | | 0 | 1 | | 0 | 0 | | 0 | 0 | |
| *Heliconia burleana* | | 0 | 0 | | 1 | 1 | | 0 | 0 | |
| *Heliconia stricta* | | 1 | 1 | | 0 | 0 | | 0 | 0 | |
| *Heliconia virginalis* | | 0 | 1 | | 0 | 0 | | 0 | 0 | |
| *Heppiella repens* | | 0 | 0 | | 0 | 0 | | 1 | 0 | |
| *Hoffmannia killipii* | | 1 | 0 | | 0 | 0 | | 0 | 0 | |
| *Kohleria villosa* | | 0 | 0 | | 0 | 1 | | 0 | 0 | |
| *Macleania bullata* | | 0 | 0 | | 0 | 1 | | 0 | 0 | |
| *Macleania pentaptera* | | 0 | 1 | | 0 | 0 | | 0 | 0 | |
| *Macleania recumbens* | | 0 | 1 | | 1 | 1 | | 0 | 0 | |
| *Macleania rupestris* | | 0 | 0 | | 0 | 0 | | 1 | 1 | |
| *Meriania tomentosa* | | 0 | 0 | | 0 | 1 | | 0 | 0 | |
| *Mezobromelia capituligera* | | 0 | 1 | | 0 | 1 | | 0 | 0 | |
| *Miconia corymbiformis* | | 0 | 0 | | 0 | 0 | | 1 | 0 | |
| *Oenothera sp.* | | 0 | 0 | | 0 | 0 | | 0 | 1 | |
| *Palicourea amethystina* | | 0 | 0 | | 0 | 0 | | 0 | 1 | |
| *Palicourea fuchsioides* | | 0 | 0 | | 0 | 0 | | 1 | 0 | |
| *Palicourea guianensis* | | 0 | 1 | | 0 | 0 | | 0 | 0 | |
| *Palicourea sodiroi* | | 0 | 0 | | 1 | 0 | | 0 | 0 | |
| *Passiflora mixta* | | 0 | 0 | | 0 | 0 | | 0 | 1 | |
| *Pernettya prostrata* | | 0 | 0 | | 0 | 0 | | 0 | 1 | |
| *Pitcairnia nigra* | | 1 | 0 | | 0 | 0 | | 0 | 0 | |
| *Pleurothallis restrepioides* | | 0 | 0 | | 1 | 0 | | 0 | 0 | |
| *Psammisia oreogenes* | | 0 | 0 | | 1 | 0 | | 0 | 0 | |
| *Psammisia sodiroi* | | 1 | 0 | | 0 | 0 | | 0 | 0 | |
| *Psychotria hazenii* | | 0 | 0 | | 1 | 0 | | 0 | 0 | |
| *Racinaea tetrantha* | | 0 | 0 | | 0 | 0 | | 1 | 0 | |
| *Renealmia aurantifera* | | 0 | 0 | | 0 | 1 | | 0 | 0 | |
| *Rubus roseus* | | 0 | 0 | | 0 | 0 | | 1 | 0 | |
| *Salvia pichinchensis* | | 0 | 0 | | 0 | 0 | | 1 | 0 | |
| *Tillandsia polyantha* | | 0 | 0 | | 0 | 0 | | 1 | 1 | |
| *Tropaeolum adpressum* | | 0 | 0 | | 0 | 1 | | 0 | 0 | |
| **Total 67** | | **14** | **13** | | **18** | **11** | | **13** | **13** | |

Table S3

Hummingbirds species detected in cameras and audiovisual census (Guevara et al., 2023). ^1^ Species analyzed in selectivity experiments

|  | | | Low | | | Mid |  | High | | | | |  |
| --- | --- | --- | --- | --- | --- | --- | --- | --- | --- | --- | --- | --- | --- |
| Hummingbird | Forest | | | Deforested | Forest | | Deforested | Forest | Deforested | Mean bill length (mm) | Mean body mass  (gr) |  |  |
| *Adelomyia melanogenys* ^1^ | | 0 | | 0 | 1 | | 1 | 0 | 1 | 14.65 | 4.31 |  |  |
| *Aglaeactis cupripennis* | | 0 | | 0 | 0 | | 0 | 1 | 1 | 17.49 | 7.77 |  |  |
| *Aglaiocercus coelestis* ^1^ | | 0 | | 1 | 1 | | 1 | 0 | 0 | 16.25 | 5.57 |  |  |
| *Amazilia tzacatl* ^1^ | | 1 | | 1 | 0 | | 0 | 0 | 0 | 19.75 | 4.89 |  |  |
| *Boissonneaua jardini* | | 0 | | 0 | 1 | | 1 | 0 | 0 | 19.20 | 8.83 |  |  |
| *Chalcostigma herrani* | | 0 | | 0 | 0 | | 0 | 0 | 1 | 13.77 | 5.30 |  |  |
| *Coeligena lutetiae* ^1^ | | 0 | | 0 | 1 | | 0 | 1 | 1 | 32.26 | 6.93 |  |  |
| *Coeligena torquata* | | 0 | | 0 | 1 | | 1 | 0 | 0 | 33.04 | 7.46 |  |  |
| *Coeligena wilsoni* ^1^ | | 1 | | 0 | 1 | | 1 | 0 | 0 | 33.57 | 6.88 |  |  |
| *Colibri coruscans* | | 0 | | 1 | 0 | | 0 | 1 | 0 | 22.28 | 6.87 |  |  |
| *Colibri thalassinus* | | 0 | | 0 | 1 | | 1 | 0 | 0 | 22.03 | 4.9 |  |  |
| *Doryfera ludovicae* | | 0 | | 1 | 1 | | 0 | 0 | 0 | 34.23 | 6.92 |  |  |
| *Eriocnemis luciani* ^1^ | | 0 | | 0 | 0 | | 0 | 1 | 1 | 19.52 | 6.00 |  |  |
| *Eriocnemis mosquera* ^1^ | | 0 | | 0 | 0 | | 0 | 1 | 1 | 18.30 | 5.70 |  |  |
| *Eutoxeres aquila* | | 1 | | 0 | 0 | | 0 | 0 | 0 | 26.68 | 10.54 |  |  |
| *Florisuga mellivora* | | 1 | | 0 | 0 | | 0 | 0 | 0 | 18.62 | 7.19 |  |  |
| *Heliangelus strophianus* | | 0 | | 0 | 1 | | 0 | 0 | 0 | 14.82 | 5.08 |  |  |
| *Heliodoxa jacula* ^1^ | | 1 | | 1 | 0 | | 1 | 0 | 0 | 21.56 | 7.46 |  |  |
| *Heliodoxa rubinoides* ^1^ | | 0 | | 0 | 1 | | 1 | 0 | 0 | 22.16 | 7.52 |  |  |
| *Heliothryx barroti* | | 0 | | 1 | 0 | | 0 | 0 | 0 | 16.50 | 5.30 |  |  |
| *Lafresnaya lafresnayi* | | 0 | | 0 | 0 | | 0 | 1 | 1 | 24.49 | 5.78 |  |  |
| *Lesbia nuna* | | 0 | | 0 | 0 | | 0 | 0 | 1 | 10.30 | 3.53 |  |  |
| *Lesbia victoriae* | | 0 | | 0 | 0 | | 0 | 1 | 0 | 14.69 | 4.96 |  |  |
| *Metallura tyrianthina* ^1^ | | 0 | | 0 | 0 | | 0 | 1 | 1 | 12.04 | 3.68 |  |  |
| *Ocreatus underwoodii* ^1^ | | 1 | | 1 | 1 | | 1 | 0 | 0 | 12.71 | 2.87 |  |  |
| *Phaethornis striigularis* ^1^ | | 1 | | 1 | 1 | | 0 | 0 | 0 | 22.63 | 2.41 |  |  |
| *Phaethornis syrmatophorus* ^1^ | | 0 | | 0 | 1 | | 1 | 0 | 0 | 39.82 | 7.03 |  |  |
| *Phaethornis yaruqui* ^1^ | | 1 | | 1 | 1 | | 1 | 0 | 0 | 40.87 | 5.81 |  |  |
| *Schistes geoffroyi* | | 1 | | 1 | 1 | | 0 | 0 | 0 | 13.73 | 3.66 |  |  |
| *Thalurania fannyi* ^1^ | | 1 | | 1 | 0 | | 0 | 0 | 0 | 19.29 | 4.19 |  |  |
| *Threnetes ruckeri* | | 0 | | 0 | 1 | | 0 | 0 | 0 | 29.93 | 6.30 |  |  |
| *Urosticte benjamini* | | 1 | | 1 | 0 | | 1 | 0 | 0 | 19.45 | 3.95 |  |  |
| **Total: 32** | | 11 | | 12 | 16 | | 12 | 8 | 9 |  |  |  |  |

Table S3. Output of structural equation models testing relationships among forest conversion into pasture (0=forest, 1= converted), morphological dissimilarity, resource abundance, and selectivity for high-elevation, mid-elevation, c) low-elevation hummingbird communities. In bold are relationships with statistical significance.

| **Model / Hypothesis** | **Estimate** | **Std.Error** | **DF** | **Crit.Value** | **P** | **Std Estimate** |
| --- | --- | --- | --- | --- | --- | --- |
| **High** |  |  |  |  |  |  |
| Morphological dissimilarity ~ forest conversion | -0.06 | 0.12 | 15.24 | 0.25 | 0.62 | -0.05 |
| **Resources ~ forest conversion** | 59.83 | 10.21 | 15.24 | 33.37 | 0.00 | **0.50** |
| Selectivity ~ morphological dissimilarity | 0.01 | 0.06 | 89.76 | 0.01 | 0.93 | 0.01 |
| Selectivity ~ resources | -0.01 | 0.00 | 74.93 | 2.69 | 0.10 | -0.17 |
| Selectivity ~ forest conversion | -0.09 | - | 104.00 | -0.92 | 0.18 | -0.10 |
| **Mid** |  |  |  |  |  |  |
| **Morphological dissimilarity ~ forest conversion** | -0.40 | 0.11 | 94.06 | 13.87 | **0.0003** | **-0.36** |
| **Resources ~ forest conversion** | 42.45 | 13.43 | 94.06 | 9.51 | **0.003** | **0.30** |
| **Selectivity ~ morphological dissimilarity** | 0.14 | 0.07 | 96.52 | 4.08 | **0.04** | **0.20** |
| Selectivity ~ resources | 0.00 | 0.00 | 96.75 | 4.08 | 0.11 | 0.15 |
| Selectivity ~ forest conversion | 0.14 | - | 103.00 | 1.45 | 0.08 | 0.14 |
| **Low** |  |  |  |  |  |  |
| Morphological dissimilarity ~ forest conversion | -0.20 | 0.12 | 76.04 | 2.74 | 0.10 | -0.17 |
| **Resources ~ forest conversion** | -40.67 | 3.53 | 75.20 | 125.70 | **0.00** | **-0.77** |
| **Selectivity ~ morphological dissimilarity** | -0.17 | 0.05 | 83.57 | 11.19 | **0.001** | **-0.24** |
| **Selectivity ~ resources** | -0.03 | 0.00 | 94.39 | 4.87 | **0.02** | **-0.19** |
| Selectivity ~ forest conversion | 0.05 | - | 98.00 | 0.51 | 0.31 | 0.05 |

Table S4. Scores from principal components analyses of hummingbird species that visited experimental feeders and enter selectivity analyses.

| **Assemblage/species** | **PC1** | **PC2** | **PC3** |
| --- | --- | --- | --- |
| **High forest** |  |  |  |
| Eigenvalues | 1.98 | 0.81 | 0.21 |
| Species scores |  |  |  |
| *Coeligena lutetiae* | 1.80 | -1.06 | -0.51 |
| *Eriocnemis luciani* | 1.10 | 0.30 | 0.30 |
| *Metalura tyrianthina* | -2.15 | -0.68 | 0.11 |
| **High converted** |  |  |  |
| Eigenvalues | 2.30 | 0.61 | 0.10 |
| Species scores |  |  |  |
| *Coeligena lutetiae* | -1.56 | -1.19 | 0.16 |
| *Eriocnemis luciani* | -1.01 | 0.66 | 0.13 |
| *Eriocnemis mosquera* | 0.90 | -0.38 | -0.55 |
| *Metalura tyrianthina* | 2.76 | -0.12 | 0.23 |
| **Mid forest** |  |  |  |
| Eigenvalues | 2.01 | 0.64 | 0.35 |
| Species scores |  |  |  |
| *Adelomyia melanogenys* | -1.20 | -0.62 | -0.001 |
| *Aglaiocercus coelestis* | -0.57 | -0.58 | 0.43 |
| *Coeligena wilsoni* | 0.98 | 0.64 | 0.18 |
| *Heliodoxa rubinoides* | 0.85 | -0.59 | 0.56 |
| *Ocreatus underwoodii* | -2.61 | -0.03 | -0.08 |
| *Thalurania fannyi* | -1.29 | 0.32 | 0.21 |
| **Mid converted** |  |  |  |
| Eigenvalues | 2.02 | 0.69 | 0.29 |
| Species scores |  |  |  |
| *Adelomyia melanogenys* | -1.19 | 0.57 | 0.12 |
| *Aglaiocercus coelestis* | -0.52 | 0.54 | -0.31 |
| *Coeligena wilsoni* | 1.07 | -0.68 | -0.02 |
| *Heliodoxa jacula* | 0.55 | 0.22 | -0.74 |
| *Heliodoxa rubinoides* | 1.04 | 0.65 | -0.32 |
| *Ocreatus underwoodii* | -2.80 | -0.20 | 0.02 |
| *Phaethornis syrmatophorus* | 1.25 | -1.28 | -0.02 |
| *Amazilia franciae* | -0.82 | -0.57 | -0.16 |
| **Low forest** |  |  |  |
| Eigenvalues | 1.96 | 0.58 | 0.46 |
| Species scores |  |  |  |
| *Heliodoxa jacula* | -1.15 | 0.82 | -0.59 |
| *Phaethornis striigularis* | 1.39 | -1.32 | 0.44 |
| *Phaethornis yaruqui* | -1.41 | -1.54 | -0.95 |
| *Thalurania fannyi* | 0.64 | -0.11 | -0.22 |
| **Low converted** |  |  |  |
| Eigenvalues | 3.02 | 0.97 | 0.01 |
| Species scores |  |  |  |
| *Amazilia tzacatl* | -1.10 | -0.38 | -0.13 |
| *Heliodoxa jacula* | 2.30 | -0.68 | 0.01 |
| *Phaethornis yaruqui* | 0.35 | 1.46 | 0.00 |
| *Thalurania fannyi* | -1.55 | -0.40 | 0.12 |
|  |  |  |  |
|  |  |  |  |
|  |  |  |  |
|  |  |  |  |
|  |  |  |  |
|  |  |  |  |

Table S5. Hummingbird species that visited experimental feeders and enter selectivity analyses. Activity at feeders is estimated as the number of visits to feeders of each species divided by the total number of visits by all species.

| **Site / Hummingbird** | **Number of visits to feeders and** | | **Activity at feeders** | **Mean ± SD time (sec) spent at low value feeder** | | **Mean ± SD time (sec) spent at high value feeder** | | | **Mean ± SD selectivity** | | | |  |
| --- | --- | --- | --- | --- | --- | --- | --- | --- | --- | --- | --- | --- | --- |
| **High forest** | |  |  |  |  | |  | | | | |  |  |
| *Coeligena lutetiae* | | 189 | 0.79 | 19.95±44.98 | | 29.05±44.87 | | 0.60±0.43 | | | |  |  |
| *Eriocnemis luciani* | | 28 | 0.12 | 18.32±19.21 | | 10.53±28.13 | | 0.34±0.44 | | | |  |  |
| *Metallura tyrianthina* | | 22 | 0.09 | 0.78 ± 1.77 | | 27.86±39.74 | 0.83±0.34 | | | | | |  |
| **High deforested** | |  |  |  | |  |  | | | | | |  |
| *Coeligena lutetiae* | | 106 | 0.23 | 57.45±99.53 | | 69.74±87.91 | 0.62±0.41 | | | | | |  |
| *Eriocnemis luciani* | | 223 | 0.47 | 67.71±107.90 | | 53.55±81.02 | 0.51±0.42 | | | | | |  |
| *Metallura tyrianthina* | | 142 | 0.30 | 70.99±116.22 | | 51.96±79.05 | 0.45±0.43 | | | | | |  |
| **Mid forest** | |  |  |  | |  |  | | | | | |  |
| *Adelomyia melanogenys* | | 1 | 0.00 | 3 | | 0 | 0 | | | | | |  |
| *Aglaiocercus coelestis* | | 62 | 0.20 | 16.63±33.00 | | 16.66±29.16 | 0.59±0.45 | | | | | | |
| *Coeligena wilsoni* | | 25 | 0.08 | 11.4±30.62 | | 25.32±33.47 | 0.62±0.48 | | | | | | |
| *Heliodoxa rubinoides* | | 68 | 0.22 | 30.81±52.34 | | 64.54±86.01 | 0.60±0.40 | | | | | | |
| *Ocreatus underwoodii* | | 136 | 0.45 | 55.44±105.12 | | 166.63±217.89 | 0.67±0.37 | | | | | | |
| *Thalurania fannyi* | | 11 | 0.04 | 83.55±89.03 | | 2.63±8.74 | 0.09±0.29 | | | | | | |
| **Mid deforested** | |  |  |  | |  |  | | | | | | |
| *Adelomyia melanogenys* | | 38 | 0.10 | 16.26±44.96 | | 35.34±54.77 | 0.62±0.48 | | | | | | |
| *Aglaiocercus coelestis* | | 122 | 0.33 | 50.88± 83.55 | | 29.63±59.94 | 0.45±0.47 | | | | | | |
| *Amazilia franciae* | | 32 | 0.09 | 0.18±0.64 | | 63.4±59.43 | 0.91±0.29 | | | | | | |
| *Coeligena wilsoni* | | 35 | 0.09 | 16.91±43.19 | | 14.94±25.81 | 0.63±0.43 | | | | | | |
| *Heliodoxa jacula* | | 26 | 0.07 | 0.04±0.19 | | 48.84±55.82 | 0.96±0.19 | | | | | | |
| *Heliodoxa rubinoides* | | 97 | 0.26 | 7.53±19.13 | | 41.92±63.68 | 0.70±0.43 | | | | | | |
| *Ocreatus underwoodii* | | 1 | 0.00 | 0 | | 8 | 1 | | | | | | |
| *Phaethornis syrmatophorus* | | 21 | 0.06 | 0.62±2.20 | | 10.81±11.23 | | | | 0.87±0.32 | |  |  |
| **Low forest** | |  |  |  | |  | | | |  | |  |  |
| *Heliodoxa jacula* | | 107 | 0.29 | 15.15±24.61 | | 18.88±38.43 | | | | 0.53±0.49 | |  |  |
| *Phaethornis striigularis* | | 25 | 0.07 | 5.12±14.31 | | 14.48±32.17 | | | | 0.61±0.46 | |  |  |
| *Phaethornis yaruqui* | | 181 | 0.49 | 29.88±62.06 | | 44.82±86.82 | | | | 0.46±0.45 | |  |  |
| *Thalurania fannyi* | | 57 | 0.15 | 19.39±57.79 | | 59.39±103.97 | | | | 0.75±0.40 | |  |  |
| **Low deforested** | |  |  |  | |  | | | |  | |  |  |
| *Amazilia tzacatl* | | 18 | 0.16 | 0.06±0.23 | | 4.5±5.4 | | | | 0.94±0.23 |  |  |  |
| *Heliodoxa jacula* | | 40 | 0.36 | 0.38±1.17 | | 10.83±16.43 | | | | 0.92±0.20 |  |  |  |
| *Phaethornis yaruqui* | | 36 | 0.32 | 2.72±4.61 | | 13.17±19.59 | | | | 0.60±0.46 |  |  |  |
| *Thalurania fannyi* | | 18 | 0.16 | 3.78±15.77 | | 3.83±4.56 | | | | 0.92±0.24 |  |  |  |

Table S6. Results of the sensitivity test applied to structural equation models testing relationships among forest conversion into pasture (0=forest, 1= converted), morphological dissimilarity, resource abundance, and selectivity for high-elevation, mid-elevation and low-elevation hummingbird communities. In this sensitivity test, morphological dissimilarity is calculated as Euclidean distance to an activity-weighted centroid (see methods). In bold are relationships with statistical significance.

| **Model / Hypothesis** | **Estimate** | **Std.Error** | **DF** | **Crit.Value** | **p** | **Std Estimate** |
| --- | --- | --- | --- | --- | --- | --- |
| **High** |  |  |  |  |  |  |
| Morphological dissimilarity ~ forest conversion | -0.10 | 0.09 | 15.24 | 1.15 | 0.30 | -0.11 |
| **Resources ~ forest conversion** | 59.83 | 10.21 | 15.24 | 33.37 | 0.00 | **0.50** |
| Selectivity ~ morphological dissimilarity | -0.05 | 0.08 | 92.67 | 0.41 | 0.52 | 0.07 |
| Selectivity ~ resources | -0.001 | 0.00 | 75.37 | 2.75 | 0.10 | -0.17 |
| Selectivity ~ forest conversion | -0.08 | - | 104.0 | -0.87 | 0.19 | -0.09 |
| **Mid** |  |  |  |  |  |  |
| **Morphological dissimilarity ~ forest conversion** | -0.44 | 0.14 | 94.06 | 9.41 | **0.002** | **-0.30** |
| **Resources ~ forest conversion** | 42.44 | 13.43 | 94.06 | 9.51 | **0.007** | **0.30** |
| **Selectivity ~ morphological dissimilarity** | 0.11 | 0.05 | 96.75 | 4.05 | **0.05** | **0.19** |
| Selectivity ~ resources | 0.001 | 0.00 | 96.67 | 2.74 | 0.10 | 0.16 |
| Selectivity ~ forest conversion | 0.13 | - | 103.00 | 1.31 | 0.10 | 0.13 |
| **Low** |  |  |  |  |  |  |
| Morphological dissimilarity ~ forest conversion | 0.02 | 0.10 | 76.04 | 0.04 | 0.83 | 0.02 |
| **Resources ~ forest conversion** | -40.67 | 3.53 | 75.20 | 125.70 | **0.000** | **-0.76** |
| Selectivity ~ morphological dissimilarity | -0.02 | 0.06 | 84.06 | 0.09 | 0.76 | -0.02 |
| **Selectivity ~ resources** | -0.003 | 0.001 | 94.67 | 6.53 | **0.01** | **-0.23** |
| Selectivity ~ forest conversion | 0.06 | - | 98.00 | 0.61 | 0.27 | 0.06 |


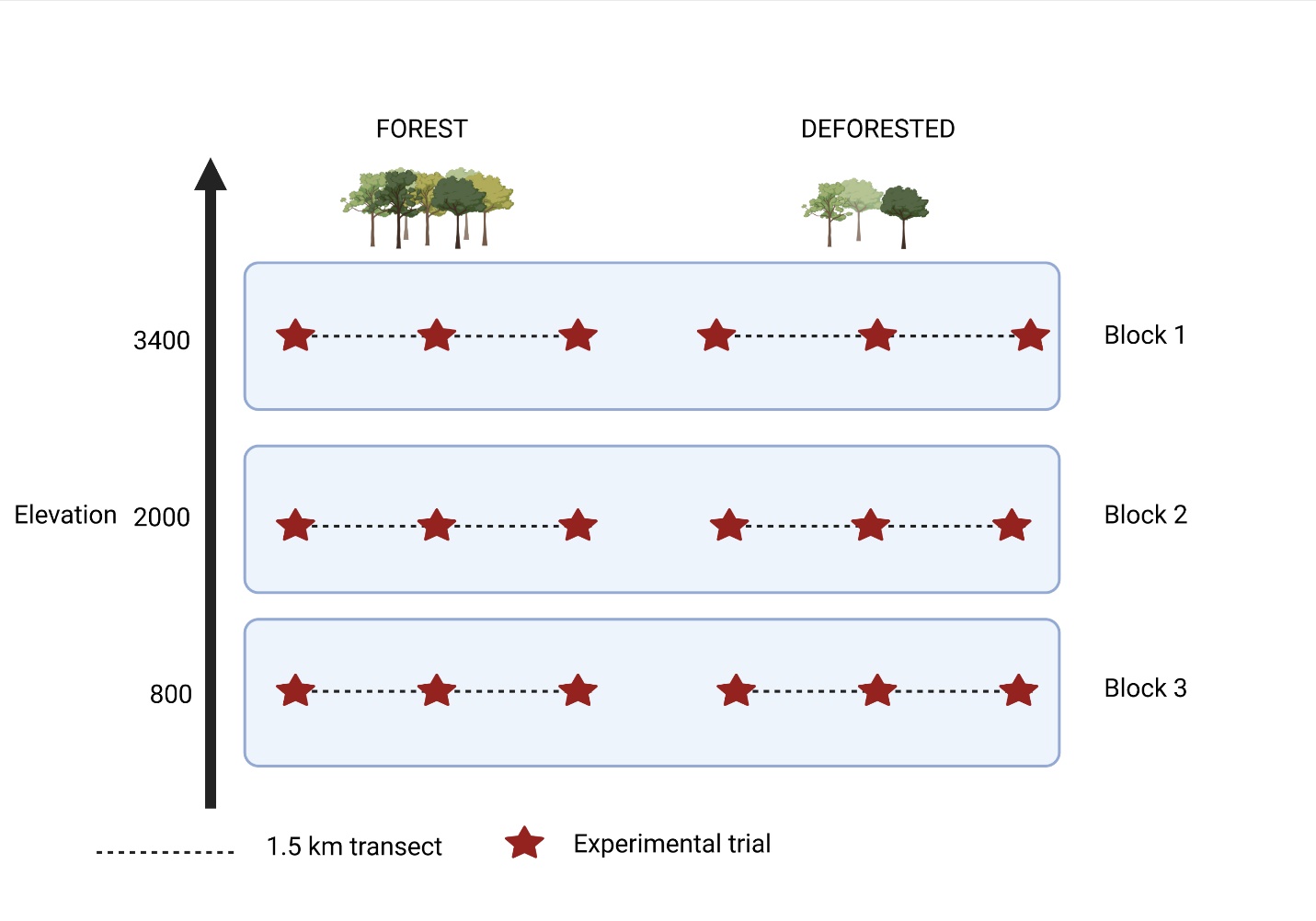


Figure S1.- Overview of the study design. Selectivity experiments were performed in three blocks spread along elevation gradient (low, mid, high). At each block three experimental trials were performed.


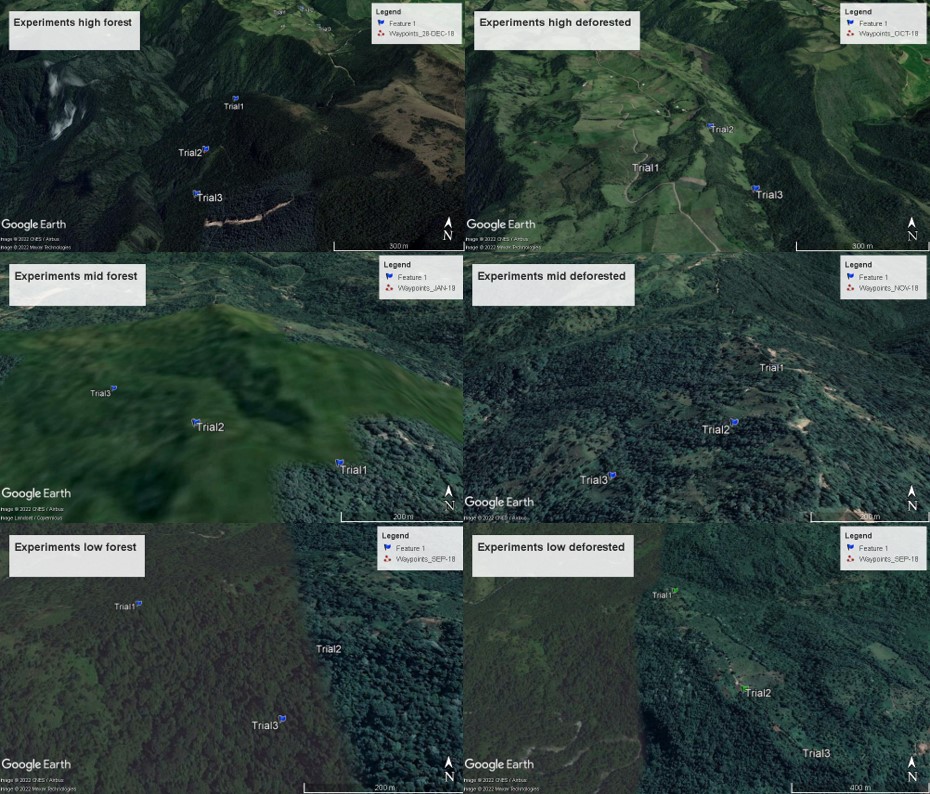


Figure S2.- Overview of the placement of experimental trials in our study area. Experimental trials were placed in areas surrounded by vegetation, in deforested sites we avoided areas completely cleared to further relate local flower resources with experimental trials.


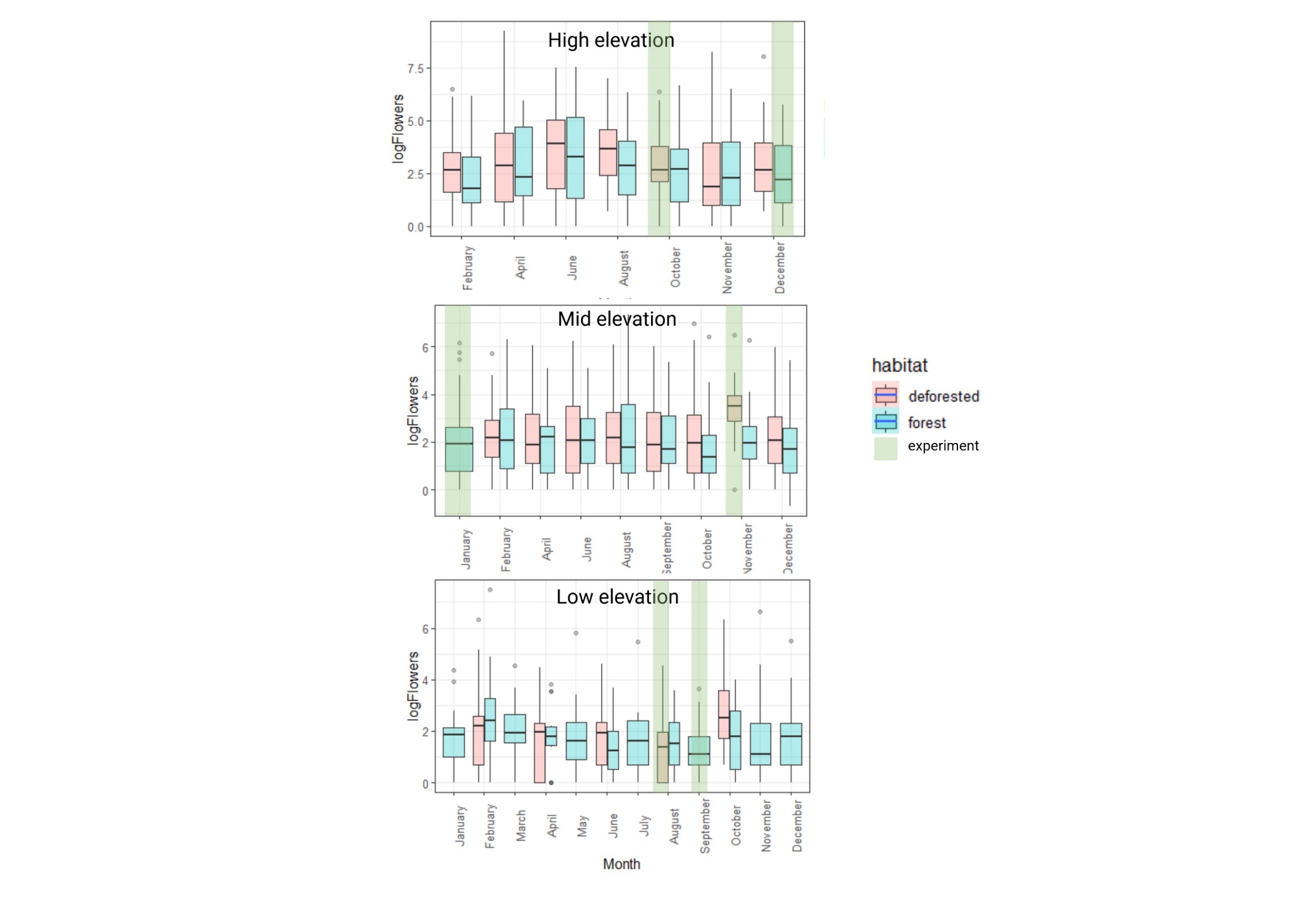


Fig S3. Seasonal and annual variation in flower abundance measured at the study sites over two-year period (Dec 2017 – Jan 2020). Boxplots reflect averaged differences in flower abundance among experimental trials. Green vertical bars indicate the month at which selectivity experiments were conducted, note that it does not indicate chronology at which experiments were performed.


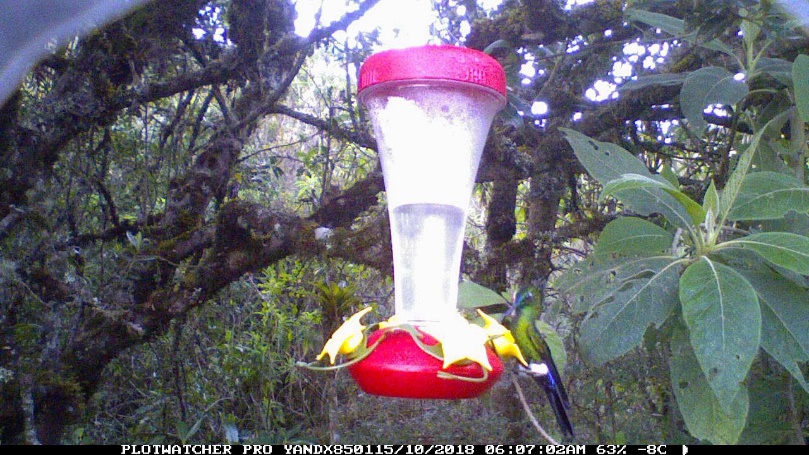

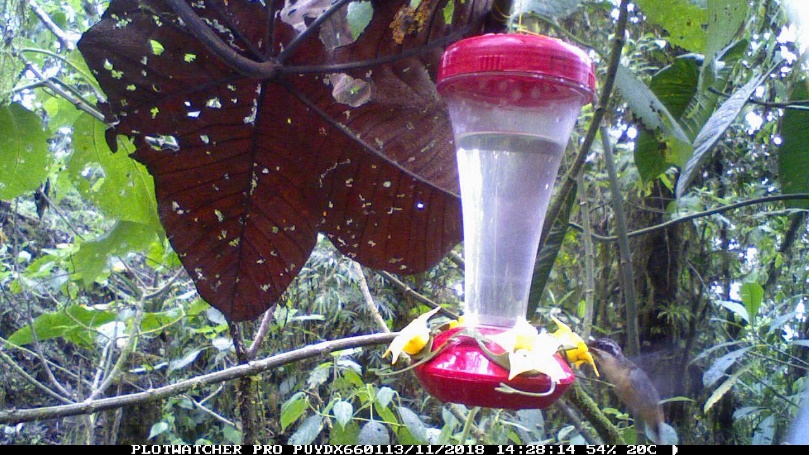


Figure S4.- Examples of images obtained by time-lapse cameras and detected with deep-meerkat motion detection software (Weinstein 2018). Left, Sapphire-vented Puffleg (*Eriocnemis luciani*) recorded at high altitude site. Right, Tawny-bellied Hermit (*Phaethornis syrmatophorus*) recorded at mid-elevation site.


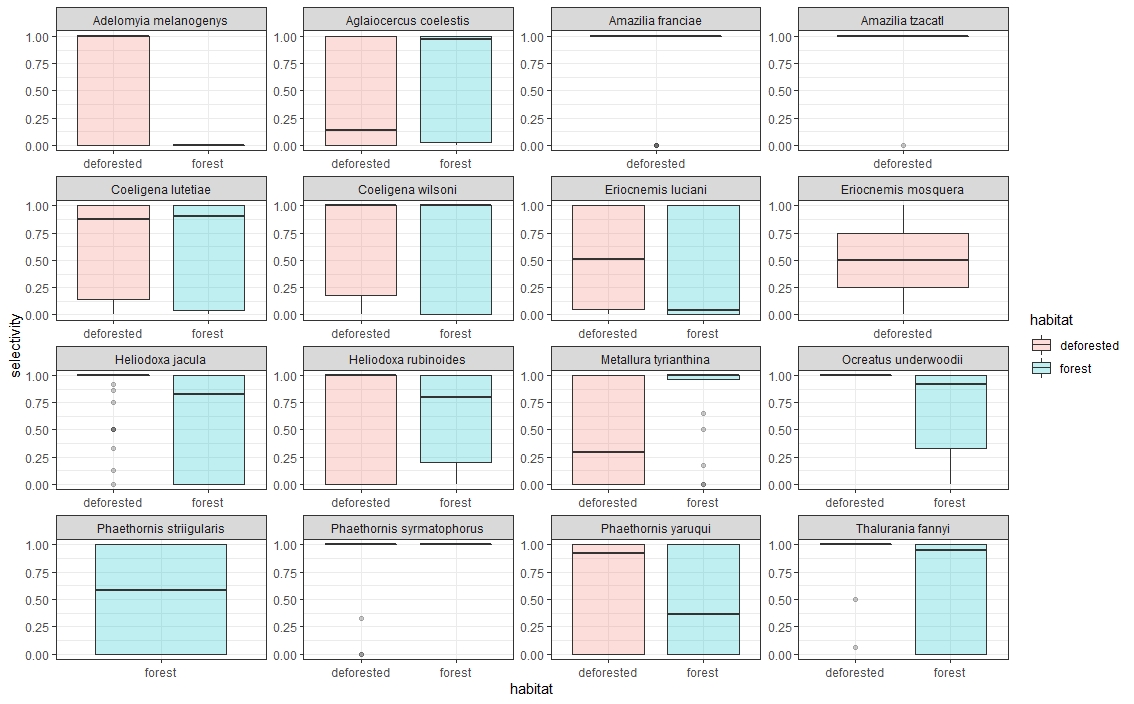


Fig S5. Differences in selectivity values for the 16 hummingbird species analyzed in this study. Twelve out of the 16 species were recorded at both forest and deforested habitats. Boxplots show
